# Targeting IL-8 and Its Receptors in Prostate Cancer: Inflammation, Stress Response, and Treatment Resistance

**DOI:** 10.3390/cancers16162797

**Published:** 2024-08-08

**Authors:** Shauna McClelland, Pamela J. Maxwell, Cristina Branco, Simon T. Barry, Cath Eberlein, Melissa J. LaBonte

**Affiliations:** 1 Patrick G Johnston Centre for Cancer Research, School of Medicine, Dentistry and Biomedical Sciences, Queen’s University Belfast, 97 Lisburn Road, Belfast BT9 7AE, UK; smcclelland11@qub.ac.uk (S.M.); pamela.maxwell@qub.ac.uk (P.J.M.); c.branco@qub.ac.uk (C.B.); 2 Bioscience Early Oncology, AstraZeneca, Cambridge CB2 0AA, UK; simon.t.barry@astrazeneca.com (S.T.B.); cath.eberlein1@astrazeneca.com (C.E.)

**Keywords:** prostate cancer, cytokines, chemokines, interleukin-8 (IL-8), CXCR1, CXCR2, inflammation, PTEN, tumour microenvironment, inhibitory combinations, small-molecule inhibitors, monoclonal antibodies, drug resistance, androgen receptor, immune response, hypoxia, PI3K/AKT pathway, clinical trials

## Abstract

**Simple Summary:**

Prostate cancer remains a significant health issue, particularly when it progresses to castration-resistant (CRPC) and metastatic CRPC (mCRPC). This review explores the role of interleukin-8 (IL-8) and its receptors, CXCR1 and CXCR2, in prostate cancer. IL-8 remains a key player in inflammation and tumour growth, promoting cancer cell survival, migration, and resistance to therapies. By targeting IL-8 signalling, researchers aim to develop new treatments that can enhance the effectiveness of existing therapies and improve outcomes for patients with resistant forms of prostate cancer. This approach could lead to better management of the disease and provide new hope for patients facing limited treatment options.

**Abstract:**

This review delves into the intricate roles of interleukin-8 (IL-8) and its receptors, CXCR1 and CXCR2, in prostate cancer (PCa), particularly in castration-resistant (CRPC) and metastatic CRPC (mCRPC). This review emphasizes the crucial role of the tumour microenvironment (TME) and inflammatory cytokines in promoting tumour progression and response to tumour cell targeting agents. IL-8, acting through C-X-C chemokine receptor type 1 (CXCR1) and type 2 (CXCR2), modulates multiple signalling pathways, enhancing the angiogenesis, proliferation, and migration of cancer cells. This review highlights the shift in PCa research focus from solely tumour cells to the non-cancer-cell components, including vascular endothelial cells, the extracellular matrix, immune cells, and the dynamic interactions within the TME. The immunosuppressive nature of the PCa TME significantly influences tumour progression and resistance to emerging therapies. Current treatment modalities, including androgen deprivation therapy and chemotherapeutics, encounter persistent resistance and are complicated by prostate cancer’s notably “immune-cold” nature, which limits immune system response to the tumour. These challenges underscore the critical need for novel approaches that both overcome resistance and enhance immune engagement within the TME. The therapeutic potential of inhibiting IL-8 signalling is explored, with studies showing enhanced sensitivity of PCa cells to treatments, including radiation and androgen receptor inhibitors. Clinical trials, such as the ACE trial, demonstrate the efficacy of combining CXCR2 inhibitors with existing treatments, offering significant benefits, especially for patients with resistant PCa. This review also addresses the challenges in targeting cytokines and chemokines, noting the complexity of the TME and the need for precision in therapeutic targeting to avoid side effects and optimize outcomes.

## 1. Introduction

Prostate cancer (PCa) remains a prevalent malignancy among men, characterized by significant diagnostic and therapeutic challenges [1,2,3]. Despite advancements in treatments, including the refinement of radiation protocols [4,5,6,7], androgen signalling inhibitors (ASIs), such as abiraterone and enzalutamide [8,9,10], and the introduction of taxane chemotherapy and polyadenosine-diphosphate-ribose polymerase (PARP) inhibitors [8,9], treatment resistance to these therapies frequently emerges [11,12]. This resistance underscores a critical need for novel therapeutic strategies targeting both the primary mechanisms of the disease and its microenvironment [4,5,6,7,8,9,10,12,13].

To address drug resistance in PCa, the use of relevant biomarkers in conjunction with clinical agents to target specific biology of advanced-stage metastatic castration-resistant prostate cancer (mCRPC) remains essential. As the knowledge of PCa has evolved, research has shifted from focusing exclusively on tumour cells to considering their dynamic interactions with the surrounding tumour and immune microenvironment (TME, IME) [14,15].

The TME plays a crucial role in PCa progression, particularly in CRPC and mCRPC [14,15]. Inflammatory cytokines and chemokines within the TME, such as interleukin-8 (IL-8), drive complex interactions to enhance tumour growth, angiogenesis, resistance to therapy, and promotion of tumour progression [16,17,18,19]. The CXCR2 signalling axis, in particular, orchestrates these interactions by modulating immune cell infiltration, angiogenic factors, and inflammation. This axis not only affects tumour cell behaviour but further shapes the TME’s overall impact on disease progression and therapeutic responses.

In addition, genetic alterations in the androgen receptor (AR), Phosphatase and tensin homolog (*PTEN*), tumour protein p53 (p53), and Transmembrane Protease, Serine 2—Erythroblast Transformation-Specific (ETS)-Related Gene (*TMPRSS-ERG*) scientifically contribute to PCa pathophysiology by altering cellular responses and treatment sensitivities [20]. In this context, exploring the CXCR1/2 axis will provide insights into the intricate dynamics between genetic drivers and the TME, offering opportunities for targeted interventions and biomarker discovery [16,17,18,19].

IL-8, a key pro-inflammatory chemokine, signals through two G protein-coupled receptors (GPCRs), CXCR1 and CXCR2 [21,22], and has been shown to be upregulated in PCa and other cancers, including breast, lung, and pancreatic [23,24,25], promoting tumour cell proliferation and migration [26]. While discussing IL-8 and its receptors, it is important to consider other ligands for CXCR1/2 that play a role in PCa, including Gro-α, Gro-β, Gro-γ, and epithelial-derived neutrophil-activating peptide 78 (ENA-78) [27]. IL-8 signalling has been linked to the transcriptional activity of AR, leading to the transition of androgen-dependent to an androgen-independent PCa [28,29]. Moreover, IL-8 driven pathways have been implicated in radiation and chemotherapeutic resistance [19]. Beyond the tumour, CXCR2-driven biology has been shown to be important in angiogenesis and in infiltrating immune cells, including neutrophils and tumour-associated macrophages (TAMs), suggesting that IL-8 may have a significant pro-angiogenic and tumourigenic role within the TME [16,26,30,31]. Given the pivotal role in enhancing tumourigenesis resistance with the TME, targeting IL-8 signalling remains a promising therapeutic approach to disrupt the tumour-, TME-, and IME-driven mechanisms of disease progression and treatment resistance.

## 2. Role and Regulation of IL-8 and CXCR1/2 in Prostate Cancer

### 2.1. IL-8: Role and Regulation

**Figure 1:**
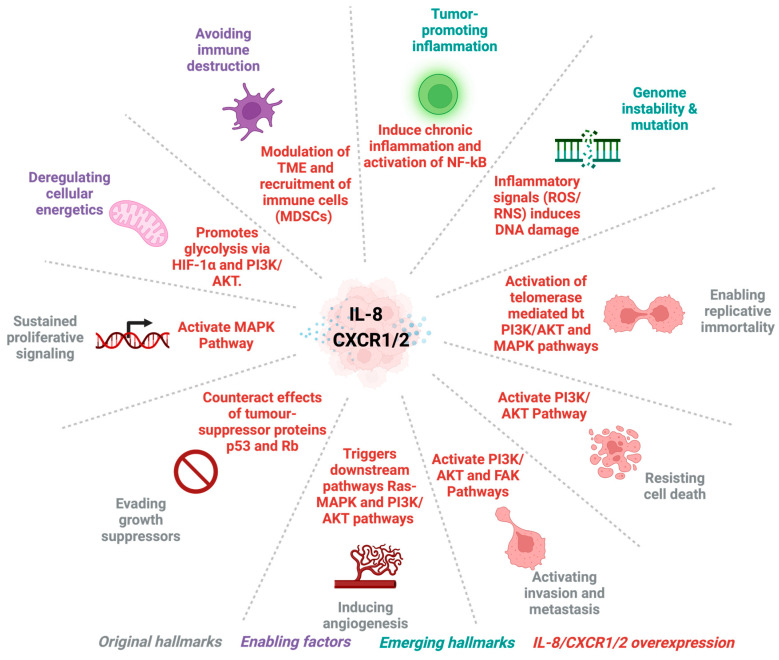
Interleukin-8 (IL-8) and CXCR1/2 signalling in cancer hallmarks. This figure illustrates the central role of IL-8/CXCR1/2 in modulating key hallmarks of cancer. IL-8, a pro-inflammatory chemokine, interacts with its receptors CXCR1/2 to exert its effect on cancer progression [39,40]. Abbreviations: CXCR1: C-X-C chemokine receptor type 1; CXCR2: C-X-C chemokine receptor type 2; FAK: focal adhesion kinase; HIF-1α: hypoxia-inducible factor-1-alpha; IL-8: interleukin 8; MAPK: mitogen-activated protein kinase; MDSC: myeloid-derived suppressor cell; NF-kB: nuclear factor kappa-light-chain-enhancer of activated B cells; p53: tumour protein P53; PI3K/AKT: phosphoinositide 3-kinase; Rb: retinoblastoma protein; RNS: reactive nitrogen species.

While IL-8 is typically expressed at low levels in healthy conditions, tumour cells often exhibit heightened IL-8 secretion in response to stimuli, including external environmental factors or treatment-mediated stress, inflammation, and/or hypoxia [16,17]. IL-8 exerts its effects through binding to its receptors CXCR1 and CXCR2, influencing various functions and regulatory mechanisms, including triggering chemotaxis, which in turn recruits cells expressing CXCR1/2 into the TME; these are primarily immune cells, including neutrophils, monocytes, and T-cells (Figure 1) [31,32,33].

**Figure 2:**
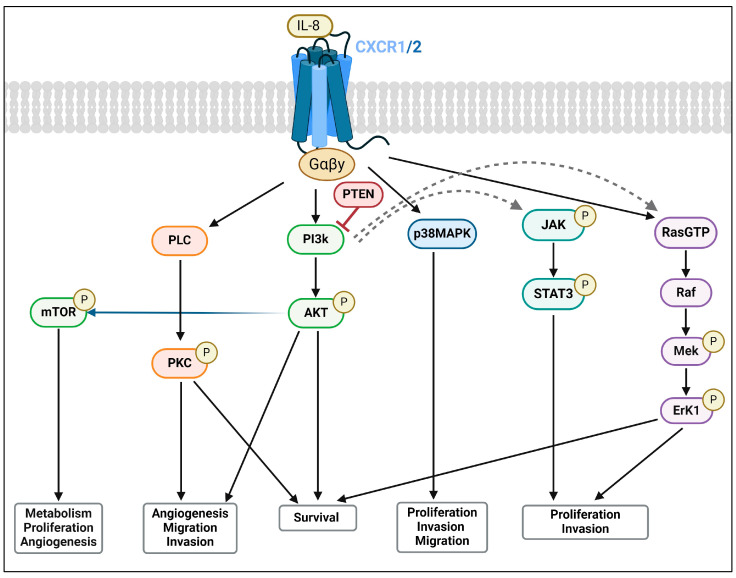
IL-8 signalling cascade and activated downstream pathways. The figure illustrates the intricate signalling network initiated by IL-8 binding to its receptors, CXCR1 and CXCR2, leading to a series of intracellular events. The cascade involves the activation of PI3K, PTEN, MAPK, AKT, and PLC, with subsequent interactions of PI3K with JAK/STAT3. The bottom of the figure highlights the linkage of the various signalling cascades to crucial cellular processes, including cell proliferation, invasion, migration, survival, metabolism, and angiogenesis. Abbreviations: AKT: protein kinase B; CXCR1: C-X-C chemokine receptor type 1; CXCR2: C-X-C chemokine receptor type 2; ERK1: extracellular signal-regulated kinase 1;IL-8: interleukin-8; JAK: Janus kinase; MAPK: mitogen-activated protein kinase; MEK: mitogen-activated protein kinase; mTOR: mammalian target of rapamycin; p38MAPK: p38 mitogen-activated protein kinase; PI3K: phosphoinositide 3-kinase; PKC: protein kinase C; PLC: phospholipase C; PTEN: phosphatase and tensin homolog; RAF: Rapidly Accelerated Fibrosarcoma; RasGTP: Ras guanosine triphosphate; STAT3: signal transducer and activator of transcription 3.

Surgery (castration) is a common treatment for PCa, and it paradoxically increases IL-8 expression in prostate epithelial cells [34]. While the molecular mechanisms underlying this response are not fully understood, the literature suggests a complex interplay between androgen signalling, inflammation, and the TME [31,34]. The abrupt reduction in androgens caused by surgery triggers an inflammatory response, activating Nuclear Factor Kappa B (NF-kB), leading to elevated IL-8 TME levels (Figure 2) [35]. This contributes to the recruitment of myeloid-derived suppressor cells (MDSCs), creating an immunosuppressive niche [34,36]. IL-8 acts as a chemoattractant for many immune cells, including neutrophils, T-cells, macrophages, and monocytes, with both pro- and anti-tumourigenic effects (Figure 1) [17,31]. While IL-8 typically recruits cells to sites of inflammation or infection to facilitate an immune response, in PCa, chronic inflammation correlates with progression to a more aggressive phenotype, leading to a constitutive influx of IL-8 [37,38].

### 2.2. CXCR1 and CXCR2: Expression and Signalling Pathways

**Figure 3:**
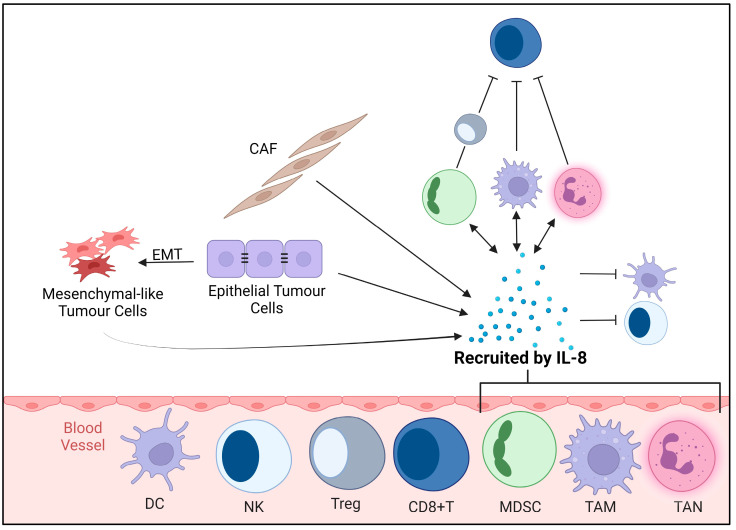
Tumour and immune response to interleukin-8 (IL-8). The figure illustrates the complex interaction of IL-8 within the tumour (TME) and immune (IME) microenvironment components. Tumour cells, CAFs, MDSCs, and TAMs all secrete IL-8, which in turn recruits MDSCs, TAMs, and TANs. IL-8 promotes tumour cell proliferation and EMT directly and indirectly. IL-8 aids in the accumulation of pro-tumourigenic immune cells and promotes immunosuppression within the TME. Abbreviations: CAF: cancer-associated fibroblast; CD8+T: CD8-positive T-cells; DC: dendritic cell; EMT: epithelial–mesenchymal transition; IL-8: interleukin 8; MDSC: myeloid-derived suppressor cell; NK: natural killer cells; TAM: tumour-associated macrophage; TAN: tumour-associated neutrophil; Treg: T regulatory cell.

IL-8 receptors have been shown to play pivotal roles in cancer progression through their expression and involvement in various pro-inflammatory, proliferative, and tumourigenic pathways, including PI3K/AKT, protein kinase C (PKC), mitogen-activated protein kinase (MAPK)/p38, RAS/ERK, and Janus kinase (JAK2), as well as signal transducer and activator of transcription (STAT3) [17]. These pathways collectively regulate cell growth, survival, and migration (Figure 2 and Figure 3) [35,41,42,43,44].

In PCa, *PTEN*-deficient cell lines have been linked to elevated CXC-chemokine signalling through CXCR1 and CXCR2 [16,27]. As GPCRs, these receptors belong to a large protein family with >800 receptors in humans; approximately 30–40% of all marketed drugs target GPCRs [45]. CXCR2 shares approximately 78% sequence homology with CXCR1, and IL-8 binds to both CXCR1 and CXCR2 with similar affinity [17,22,46].

Murine models have been invaluable tools when studying PCa. However, there are inherent limitations when it comes to investigating specific molecular pathways such as the IL-8 signalling axis. One significant challenge is the disparity between mouse and human chemokine receptors, CXCR1 and CXCR2 (Table 1). While mice express functional CXCR2, evidence suggests that the murine genome lacks a direct ortholog for human CXCR1 [47,48,49]. In addition, murine orthologs lack the gene coding for IL-8 (*CXCL8*); instead, they express KC (*CXCL1*), MIP-2 (*CXCL2*), and Gro (*CXCL1*), which bind to the murine functional CXCR2 receptor but do not fully replicate the function of human IL-8 [46,47,50]. These differences limit the translatability of findings from murine models to human disease, particularly when targeting IL-8 and its receptors for therapeutic intervention.

To address these limitations, various strategies have been employed in other disease models that can be adapted and translated to PCa studies moving forward. Using genetically engineered mouse models (GEMMs), a human CXCR1 knock-in (hCXCR1 K1) mouse model has been developed, where the transgene is under the control of native mouse promoter and regulatory elements [51]. This model expresses hCXCR1 at levels comparable to those observed in human neutrophils, providing a valuable tool to investigate the therapeutic efficacy of small molecules or monoclonal antibodies targeting CXCR1 [51]. Specifically, to address IL-8 expression in murine models, mice carrying a bacterial artificial chromosome encompassing the entire human IL-8 (*CXCL8*) gene, including its regulatory elements, have been developed (IL-8Tg) [49]. These IL-8Tg mice strongly express human IL-8 upon inflammatory stimulation, leading to an increased mobilization of immature myeloid cells and enhanced tumourigenesis in a colorectal cancer model [49]. Beyond GEMM models, patient-derived xenografts (PDXs) can be adapted for studies to retain the heterogeneity and complexity of human tumours, providing a robust platform for studying the tumour-intrinsic modulation of the IL-8/CXCR1/2 axis in PCa, but they are limited in their application to the murine immune compartment [52,53,54]. Organoids, 3D cultures derived either from PCa cell lines or patient tumours that maintain the genetic and phenotypic characteristics of the original tumour, may offer a promising platform for drug screening and understanding IL-8/CXCR1/2 tumour-intrinsic and TME biology in a controlled environment [55]. Recent advancements have integrated co-culture systems where organoids grow alongside human immune cells, such as T-cells and macrophages, to better replicate the TME and study the interactions between cancer cells and the immune system [56]. These co-culture models can be used to investigate the role of IL-8 in tumour progression, immune cell recruitment, and therapeutic resistance in PCa.

Numerous studies have investigated the molecular mechanisms underlying the inhibition of IL-8 or its receptors, revealing a multifaceted impact on PCa cells [57,58]. Notably, in *PTEN*-deficient PCa cells, the inhibition of CXCR1/2 induces a reduction in CXC-chemokine signalling. Indeed, preclinical investigations in the context of *PTEN* loss have shown that targeting IL-8 or its receptors enhances the sensitivity of PCa cells to ionizing radiation [28], indicating that the potential of this approach may depend on specific genetic contexts. While in vitro experiments provide valuable insight into the potential therapeutic effects of targeting CXCR1/2, the lack of in vivo validations limits the representation of the TME and immune infiltrates. Clinical evidence showed that targeting CXCR2 in combination with enzalutamide re-sensitized patients who had previously developed resistance to ASIs [59], highlighting the potential synergy with existing therapeutic modalities. Below is an in-depth exploration of the molecular intricacies and therapeutic efficacy associated with inhibiting IL-8, CXCR1, and CXCR2 in PCa, shedding light on the promising strategies for providing alternative approaches to combating this complex disease [60].

## 3. Impact of IL-8/CXCR1/2 on the Tumour Microenvironment

The intricate crosstalk of IL-8 and its receptors with the TME plays a pivotal role in cancer progression [31]. IL-8 orchestrates a dynamic interplay within the TME by engaging with CXCR1/2. The autocrine and paracrine modulation of IL-8 fosters tumour-associated inflammation [17,46,61], the epithelial–mesenchymal transition (EMT), and neovascularization [62]. Dysregulated IL-8 signalling profoundly influences immune cell recruitment, promotes MDSC infiltration, and modulates the immune landscape [32]. The crosstalk between IL-8 and its receptors contributes to the spatial and temporal heterogeneity within the TME, influencing both the pro-tumourigenic and anti-tumourigenic effects mediated by immune cells [63,64]. Understanding the complex interactions between IL-8, CXCR1, and CXCR2 within the TME is crucial for developing targeted therapeutic strategies for PCa and addressing tumour progression, dissemination, and treatment resistance.

### 3.1. Vascular Dynamics and Cancer Cell Communication

In PCa, hyperactivation of the IL-8/CXCR1/2 signalling axis stimulates EC (endothelial cell) proliferation, migration, and tube formation. This process promotes the formation of new blood vessels, which in turn sustains the growing tumour with nutrients and oxygen [37]. This has also been seen in immunohistochemistry analysis of human PCa biopsy samples [65]. Angiogenesis, driven, in part, by the IL-8/CXCR1/2 signalling axis, involves downstream signalling pathways such as the PI3K/AKT and MAPK cascades. Targeting this axis provides a promising therapeutic strategy to modulate the TME, inhibit angiogenesis, and reduce PCa progression.

Cancer cells within the TME are not isolated; they actively engage, sense, and interact with the surrounding non-cancerous cells. These interactions play a key role in facilitating various aspects of tumour progression, including growth, invasion, angiogenesis, and immune evasion [66]. In normal prostate tissue, vascular perfusion involves coordination between ECs and perivascular cells, mainly pericytes and smooth muscle cells, ensuring stable and functional blood vessels. In contrast, in PCa tumours, the architecture is disrupted, leading to the formation of abundant, immature, unstable, and leaky blood vessels [67]. Abnormal leaky vasculature results in poor perfusion and hypoxia, which in turn stimulates the secretion of pro-angiogenic factors such as vascular endothelial growth factor A (VEGF-A) and pro-inflammatory cytokines including IL-8 [61], further stimulating the accumulation of neutrophils and macrophages and exacerbating tumour progression and treatment resistance [66].

Research by Zhao et al. has shown that ECs contribute to metastatic potential in PCa by suppressing AR expression and activity [68]. This indicates that ECs not only form the blood vessel structure, but also actively participate in hormonal dynamics [35], influencing PCa progression and offering potential targets for disrupting these critical interactions within the TME [68,69]. Early studies focusing on chemotherapy combinations often used sensitivity shift or clonal recovery in vitro assays. However, these studies did not include biomarker work to differentiate or confirm direct versus TME-mediated effects, which can now be observed with in vivo studies [70,71,72]. Thus, translating these findings to in vivo proof-of-concept studies was limited, potentially overlooking the role of neutrophils, which were not well understood at the time [73].

#### Cancer Stem Cell-Mediated Tumour Progression and Treatment Resistance

Cancer stem cells (CSCs), also known as tumour-initiating cells, are essential in the onset of metastasis in almost all cancer types. The IL-8/CXCR2 axis may have an important role in tumour progression and invasion by maintaining and promoting the migration of CSCs [24]. ECs surrounding CSCs have been shown to not only secrete IL-8 but also upregulate CXCR2 expression in CSCs, which enhances CSC migration and growth [74]. Some studies have suggested that the activation of CXCR2 and/or increases in IL-8 promote stem-like properties in cancer cells, including enhanced self-renewal and resistance to differentiation [74,75]. One of the significant challenges in treating PCa is the development of treatment resistance [76]. CSCs are thought to be responsible for the onset of resistance due to their inherent properties, such as quiescence and enhanced DNA repair mechanisms [76,77,78]. As a result, conventional treatments that target rapidly dividing cancer cells, such as radiation, hormone therapy, and chemotherapy, may not completely eliminate CSC populations, allowing for tumour recurrence [31]. Therefore, IL-8 inhibition can be an avenue to address the reversal or limitation of the accumulation of CSCs in PCa that contributes to treatment resistance and disease progression.

### 3.2. Immunosuppressive TME

Myeloid cells are essential in both the innate and adaptive immune response [31]. The TME has the ability to change myeloid cells into immunosuppressive cells. Neutrophils and MDSCs are two major myeloid-derived cells that foster the interaction between cancer cells and the IL-8/CXCR1/2 axis. IL-8 attracts these immune cells, which can dampen the immune response and allow tumour cells to persist and progress [79]. MDSCs, a group of immature immunosuppressive cells, expand during cancer, inflammation, and infection, where they function to suppress an anti-tumourigenic T-cell response [80]. It is still not clear from the literature whether MDSCs are the prerequisite for tumour progression or if a progressing tumour recruits MDSCs. Some studies suggest that MDSCs play a role in aiding tumour progression and that their presence inhibits the anti-tumour activity of immune cells, allowing the tumour to survive and progress by creating an immunosuppressive microenvironment [80,81,82]. In contrast, other research suggests that tumours actively recruit MDSCs by releasing various factors, including IL-8 [83,84]. It is important to understand the directionality of MDSCs to aid in therapeutic targeting. Targeting IL-8 could aid in modulating their function and/or inhibit their recruitment, as well as enhance immunotherapy strategies.

### 3.3. Modulation of Immune Response and Inflammation

Inflammation remains an important hallmark of cancer, and in PCa, chronic inflammation has been associated with disease initiation and its progression to a more aggressive phenotype and treatment resistance [73]. Within the TME, there are many responses which occur due to chronic inflammation, including the establishment of an inflammatory microenvironment that is characterized by the infiltration of immune cells (macrophages, neutrophils, and T-cells), cytokines (IL-8, IL-6, TNF-α, and IL-1β) and chemokines [15,73]. IL-8/CXCR1/2 inhibition has shown evidence suggesting that targeting this pathway can modulate chronic inflammation. For instance, studies have shown that inhibition of IL-8/CXCR1/2 can reduce the recruitment of immune cells and decrease the levels of pro-inflammatory cytokines, thereby potentially mitigating the inflammatory environment within the TME and reducing tumour progression and treatment resistance [34,59,85].

### 3.4. Cancer Cell Plasticity: Induction of Epithelial–Mesenchymal Transition (EMT) and Metastasis

The interaction between IL-8 and its receptors CXCR1/2 can promote tumour cell survival through the EMT, which enhances the migratory ability of tumour cells. During the EMT, epithelial cells alter their characteristics by acquiring mesenchymal-like properties, often linked to an increase in cell migration, invasion, and metastasis [86]. The EMT can be regulated directly and indirectly by multiple molecular mechanisms, including growth factors and cytokines such as transforming growth factor-beta (TGF-β), epidermal growth factor (EGF), and insulin-like growth factor (IGF), and signalling pathways such as MAPK and PI3K/AKT [86,87]. These signalling pathways in turn can induce expression of transcription factors like snail, slug, twist, and Zeb1/2, which are known regulators of the EMT [88].

Various markers associated with the EMT have been reported in PCa patient tissues; for example, UBE2R2-AS1, a long non-coding RNA, has been reported to be significantly upregulated in CRPC and mCRPC and has been linked to a high Gleason score, metastasis, and poor prognosis [89]. The histone demethylase KDM5C has been shown to be highly expressed in mCRPC and its knockdown reduces migratory and invasive capacity, potentially through modulation of the EMT signalling pathway [90].

Overall, the signalling induced by IL-8 on either CXCR1 or CXCR2 influences many aspects of tumour progression within the TME, providing promising targets for therapeutic interventions. Inhibiting these interactions could limit tumour growth, angiogenesis, and metastasis. Specifically, IL-8/CXCR1/2 signalling has been linked to promoting the EMT [43,91]. There are several inhibitors developed that target IL-8 or its receptors CXCR1 and/or CXCR2 (Table 2), with several ongoing clinical trials in PCa targeting these pathways to evaluate their effectiveness (summarized in Table 3). By targeting this pathway, it may be possible to inhibit the EMT, thereby reducing metastasis burden and improving clinical outcomes.

## 4. TME and Its Significance in Cancer

The prostatic stromal microenvironment contains multiple elements that are anatomically and physiologically essential to the normal functioning of the gland [92]. However, alterations within these stromal factors contribute significantly to the development, progression, and metastasis of prostate cancer, as well as to the therapeutic response [14,26]. The TME is a complex and dynamic network involving interactions between tumour and non-cancerous cells and components. Additionally, soluble factors like cytokines and chemokines play critical roles in responding to environmental cues and tumour pathology [35,93,94,95]. The interplay between these elements not only supports tumour growth and survival but also mediates resistance to therapies and contributes to immune evasion [81,82]. Understanding these interactions is crucial for developing new therapeutic strategies targeting the TME to improve patient outcomes.

### 4.1. ECM

Surrounding prostate epithelial cells is a tightly interlocking extracellular matrix (ECM), which actively engages and interacts with PCa cells. It is composed of collagen and multiple non-collagenous proteins such as bone sialoprotein (BSP), fibronectin, laminin, and cadherins [96]. The ECM, a critical component of the TME, provides a physical scaffold for cells, constituting up to 60% of tumour mass, and plays a major role in promoting cancer cell progression [97]. In PCa, the ECM is often remodelled, resulting in increased stiffness, and altered composition, impacting cancer cell motility and increased invasive behaviour through the induction of EMT and subsequent metastasis [98].

Several physical and chemical factors significantly influence the ECM. Interstitial pressure within the TME can impact vascular function, exacerbate hypoxia, and hinder the delivery of nutrients and oxygen to cancer cells, thereby complicating treatment delivery and response [97]. Additionally, the TME often exhibits a lower extracellular pH (acidosis) due to poor perfusion and tumour vasculature. Acidosis can impact cellular processes including proliferation, invasion, and therapy resistance [97,99]. These changes in the ECM environment directly impact IL-8 and CXCR signalling pathways, which are crucial for PCa progression.

Cancer-associated fibroblasts (CAFs) within the TME are the most predominant source of ECM components, including matrix metalloproteinases (MMPs), which degrade ECM proteins and facilitate the release of IL-8 and growth factors such as VEGF-A, fibroblast growth factors (FGFs), platelet-derived growth factor (PDGF), and TGF-β. These factors promote angiogenesis, tumour progression. and metastasis [100]. In the context of PCa, IL-8-mediated CXCR2 signalling on cancer cells, ECs, and immune cells enhances these processes, further supporting tumour growth, metastasis, and treatment resistance.

### 4.2. Heterogeneity

PCa exhibits both spatial and temporal heterogeneity with the TME, differing across various regions of the tumour, as well as at different stages of cancer progression [101]. Additionally, the TME can adapt in response to treatments, further complicating the PCa landscape [94,102].

Intertumoural heterogeneity is marked by variations in cell type, gene expression, and microenvironmental conditions within different areas of the same tumour [103,104]. This spatial heterogeneity can be seen, for example, in the distinct differences between tumour margins and central regions. Tumour margins often resemble normal tissue, with balanced composition and lower stiffness, facilitating cell–ECM interactions and immune surveillance. In contrast, the core tumour region exhibits altered ECM composition, increased stiffness, and fibrosis, promoting tumour progression, angiogenesis, and immune evasion [104,105]. This central area is critical for invasion and metastasis and often harbours tumour-promoting cells, including CAFs, and cytokines, including IL-8 [106,107].

Temporal heterogeneity refers to changes in the TME that occur over time as the disease progresses. Early stages of PCa are characterized by a less hypoxic and inflammatory environment compared to later stages, where oxygen is limited, and inflammation is more pronounced [108]. Advanced stages of PCa often involve significant genetic alterations, such as loss of *PTEN* or mutation/deletion of TP53 [106]. Moreover, treatments like radiation and androgen deprivation therapy (ADT) can also alter the TME, contributing to further heterogeneity and evolution by increasing inflammation, changing vasculature structure and driving of tumour cell resistance [109,110].

Understanding the variability of the TME throughout disease progression and the implementation of treatment approaches is crucial for the development of new treatment strategies. This understanding allows for a more tailored treatment regime based on genetic signatures such as AR responsiveness, hypoxic signature, or DNA damage signature [111,112,113].

### 4.3. Inflammation

Cancer-related inflammation, a hallmark of cancer, has been shown to support the growth, survival, invasion, and migration of tumour cells [38]. Chronic inflammation in PCa is associated with high-grade tumours [114], and persistent inflammation within the prostate leads to the influx of multiple immune cells such as macrophages, natural killer (NK) cells, and mast cells, creating a pro-tumourigenic TME [38,73]. Inflammatory cells release high amounts of cytokines and chemokines that promote tumour growth and metastasis, including tumour necrosis factor (TNF), NF-kB, IL-6, IL-8, and VEGF-A [15,73].

### 4.4. Immune Response

The immune system exhibits a dual role in cancer. It can suppress tumour growth through immune surveillance via tumour-infiltrating lymphocytes (TILs), such as cytotoxic T-cells and NK cells, recognizing and eliminating cancer cells [115]. On the other hand, tumours often evade immune attack through fostering an immunosuppressive microenvironment (IME), as seen in PCa. The immunosuppressive nature of the prostate involves cellular and molecular mechanisms that collectively enables tumour survival and growth while limiting the immune system’s ability to recognize and eliminate cancer cells [116]. PCa is often referred to as an “immune-cold tumour” due to its low tumour immunogenicity, resulting in a very limited response to immunotherapies [116,117]. The PCa IME impacts anti-tumour immune responses via the upregulation of immune checkpoint proteins (e.g., PD-L1) to inhibit T-cell activity [118,119], increasing the expression of IL-8 [117] and the secretion of growth factors, facilitating angiogenesis and matrix remodelling from TAMs, and promoting tumour growth and immunosuppression to drive tumour progression [61].

Immunotherapies that block immune checkpoint molecules have demonstrated success in reinvigorating anti-tumour immune responses in PCa and other cancers including melanoma, lung, bladder, colorectal, and triple-negative breast [120,121,122]. The most promising immune checkpoint inhibitors to be FDA-approved are anti-CTLA-4, anti-PD-1, and anti-PD-L1 monoclonal antibodies [123], but their clinical application and effectiveness is still being explored in combination with other therapies to investigate their effectiveness in enhancing the outcomes for patients. Combinational therapies targeting multiple immune checkpoint inhibitors in conjunction with current therapies (radiation, ADT, olaparaib, docetaxel) may be required for an effective response in PCa [110,124]. Current studies investigating checkpoint inhibitors have shown that Herpesvirus Entry Mediator HVEM (also known TNF Receptor Superfamily Member 14, or TNFRSF14), expressed on several cell types, including T-cells, B-cells, NK cells, and myeloid cells, has shown promising results as an immune checkpoint for T-cell mediated tumour control in PCa [125]. Prostate-specific membrane antigen (PSMA) targeting radionuclide therapy (RNT) in combination with anti-PD-1 has also shown promising therapeutic results in a mouse model of PCa (C57BL/6-mice bearing syngeneic RM1-PGLS tumour) [126,127]. It is important to consider the complex TME and IME interactions when designing combination therapeutic approaches to effectively combat cancer and enhance patient outcomes. IL-8 is implicated in multiple interactions within the TME and IME, highlighting the complexity of targeting this axis.

## 5. Therapeutic Implications and Challenges

### 5.1. Therapeutic Implications

There are ongoing clinical trials to understand the benefit of targeting chemokine receptors in PCa (Table 3). Inhibiting IL-8 interactions with CXCR1/2 has been shown to have a therapeutic benefit in clinical trials in combination with enzalutamide in treatment-resistant PCa (Expanded in Section 5.4) [59]. Challenges remain regarding the therapeutic inhibition of CXCR1/2, raising two important questions: (1) Do we need to inhibit both CXCR1 and CXCR2 in prostate cancer to see a therapeutic benefit? (2) Would patients benefit from targeting CXCR1, CXCR2, IL-8, or a combination of these in conjunction with existing treatments, particularly for those who have developed resistance to current therapies [16,85]? As illustrated in lung diseases like asthma and COPD, CXCR2 appears to be the most important pathologically; however, this has not been confirmed in PCa and more research is required [128,129].

### 5.2. Therapeutic Inhibition of CXCL8/CXCR1/CXCR2: Small-Molecule Inhibitors, Antagonists, and Monoclonal Antibodies

The therapeutic inhibition of IL-8 and its receptors, CXCR1/CXCR2, shows promise across a range of diseases. As previously mentioned, chemotherapy drives IL-8 expression and secretion in numerous cancers [79,130,131] and its direct inhibition in vitro has been shown to enhance efficacy to radiation, hormone, and chemotherapy. In preclinical models of human hepatocellular carcinoma (HCC), cancer cells treated with cisplatin and doxorubicin upregulated IL-8 expression, but, importantly, that study demonstrated that the pre-treatment of the cancer cells with an siRNA that specifically silenced IL-8 before cisplatin/doxorubicin treatment significantly increased treatment efficacy [132]. Clinical data treating multiple solid tumours, including PCa, by targeting IL-8 using HuMax-IL8 (a monoclonal Ab targeting IL-8) showed that inhibition of IL-8 led to a significant reduction in serum IL-8 levels, which was associated with enhanced therapeutic responses [133]. The study indicated potential synergy with other therapies, though it primarily focused on safety and pharmacokinetics in a Phase 1 setting. There was an overall response (15 patients) showing stable disease observed in 73.3% of patients, with a progression-free survival rate of 53.3% at 5.5 months. The focus on IL-8 inhibition has evolved to the inhibition of its receptors CXCR1 and CXCR2, particularly as their expression has been shown to increase following radiation exposure and disease progression, suggesting that targeting these receptors might be more effective than targeting IL-8 directly.

In cardiovascular disease models, CXCR1/2 inhibition has demonstrated substantial benefits, including atherosclerotic plaque reduction and improved lipid profiles [134]. In pancreatic cancer, inhibiting CXCR1/2 with SC-479833 demonstrated anti-tumour and anti-metastatic effects, along with decreased neutrophil recruitment [135]. Additionally, studies have shown that neutrophil recruitment plays a significant role in liver metastasis rather than the primary tumour in pancreatic cancer [124,136], and similar findings have been observed in colon cancer [137] and HCC [138], highlighting the context-dependent nature of CXCR2’s role. Focusing on PCa, therapeutic inhibition of IL-8 or its receptors CXCR1 and CXCR2 demonstrated reduced tumour growth and increased treatment sensitivity in resistant tumours [59,85]. Interestingly, reports indicate that CXCR2 overexpression enhances prostate tumourigenesis, while CXCR1 overexpression inhibits it [135,139]. CXCR1 overexpression also blocked AKT activation and signal transduction, suggesting that the selective inhibition of CXCR2, rather than the inhibition of IL-8 binding to CXCR1 and CXCR2, is more important in PCa [139]. A major challenge with the inhibition of IL-8 remains sustaining its inhibition due to its high levels and rapid turnover as a ligand, which poses a challenge for effective long-term therapy [30].

### 5.3. Combinational Therapies and Emerging Treatment Modalities

For PCa patients, receiving ADT, while initially effective, often leads to resistance within 2–3 years, with their disease progressing into CRPC/mCRPC [76,140]. The current treatment of mCRPC uses ADT in combination with other agents, including docetaxel, cabazitaxel, or radionuclide radium 223, to target bone metastasis [141,142]. Despite the benefit and enhanced survival rate when used in sequence (median overall survival ranging from 21 to 29 months), there are challenges in treatment sequence optimization and resistance development [143,144]. With the lack of curative treatment options for mCRPC, various immunotherapies, including anti-CTLA-4 and anti-PD-1 treatments, have been clinically evaluated, with limited efficacy [145,146].

Recent studies suggest that a combination of CXCR2 inhibition in combination with standard-of-care treatment enzalutamide may increase survival for mCRPC, suggesting that interfering with cytokine and chemokine pathways by focusing on the TME and IME could be more beneficial to patients [59,147]. This approach aligns with the finding that IL-6 and IL-8 are critical in androgen-independent AR signalling [64,148,149]. The inhibition of CXCR2 using AZD5069 attenuated MDSC-mediated castration resistance and extended the anti-tumour effect of enzalutamide [150], which has also been investigated through the Phase 1/2 ACE trial (see Section 5.4).

### 5.4. Clinical Trials

The ACE trial (NCT03177187) evaluated the efficacy of the CXCR2 inhibitor AZD5069 in combination with enzalutamide in mCRPC patients [59]. Of twenty-one patients, five had a partial response, as evidenced by decreased prostate-specific antigen (PSA), reduced measurable disease, and extended radiological progression-free survival [59]. This combination also notably reduced myeloid inflammation, a negative prognostic marker in PCa, highlighting the potential of therapy targeting CXCR2 in combination with standard treatments [59].

Another Phase I trial used HuMax-IL8 (BMS-986253; NCT02536469), an anti-IL-8 monoclonal antibody, in patients with metastatic or unresectable solid tumours, including PCa. This study was conducted to investigate the maximum tolerated dose and assess safety profiles. Fifteen patients were enrolled, and the study included both dose-escalation and dose-expansion phases. There were no dose-limiting toxicities and no severe adverse effects related to HuMax-IL8. Pharmacokinetic analysis revealed dose-proportional increases in drug exposure. The best overall response was stable disease observed in 73.3% of patients, with a progression-free survival rate of 53.3% at 5.5 months [133].

Currently recruiting is a clinical trial Synergy-201 (NCT06228053), investigating SX-682, an inhibitor of CXCR1/2, in combination with enzalutamide in men with abiraterone-resistant metastatic castration-resistant PCa. Another trial which is ongoing (MAGIC-8; NCT03689699) is a Phase 1b/2 trial evaluating the safety and efficacy of nivolumab alone or combined with BMS-986253 (IL-8 monoclonal antibody) plus androgen deprivation therapy in men with hormone-sensitive prostate cancer.

## 6. Challenges in Implementing Therapies Targeting IL-8-CXCR1/2 Axis for PCa Treatment

### 6.1. Development of Treatment Resistance

The evolution of CRPC/mCRPC post-ADT intervention is a testament to the adaptive capacity of PCa [76,151]. The mechanisms of drug resistance in CRPC are complex and diverse, involving many pathways that have been described and/or are linked to the development of CRPC/mCRPC [152]. They often involve AR signalling alterations [76,151,153,154], lineage plasticity [140,155], and genetic mutations, including *PTEN* loss [112], resulting in the activation of survival pathways like PI3K/AKT/mTOR (Figure 3) [147,156]. These pathways, influenced by IL-8 and its receptors [157,158], contribute to the resilience of PCa against conventional therapies, challenging the development of lasting therapeutic responses (Figure 2) [16].

Specifically, in PCa, IL-8 signalling directly contributes to drug resistance by activating survival pathways and inhibiting apoptosis. Specifically, IL-8 promotes the activation of the PI3K/Akt pathway and increases the transcription of anti-apoptotic proteins such as Bcl-2 and survivin through the CXCR2/NF-kappaB signalling axis [64,159]. This mechanism is particularly relevant to the resistance observed with chemotherapeutic agents like oxaliplatin in metastatic PCa cells [160]. Furthermore, IL-8 can regulate the transcriptional activity of AR, thereby supporting androgen-independent proliferation and resistance to androgen receptor-targeted therapies [26,28].

Novel therapeutic strategies aiming at co-inhibiting chemokine signalling (e.g., IL-8/CXCR1/2) alongside standard therapies show promise in enhancing treatment sensitivity and countering resistance by countering tumour-intrinsic and TME- and IME-mediated mechanisms.

### 6.2. Specificity and Efficacy of CXCR1/2 Targeting

Despite the potential of CXCR1/2 inhibitors, their expression on cells beyond those of the tumour leads to specificity challenges and possible off-target effects. CXCR1 and CXCR2 are also found on many other cell types, including epithelial, endothelial, tumour, and stromal cells [58,161]. Dual inhibition strategies targeting these receptors in tumour cells and in the TME and IME may offer improved outcomes by disrupting tumour growth and immune evasion. However, the variable expression and role of CXCR1/2 necessitates a nuanced approach, tailored to tumour characteristics and disease stage to maximize therapeutic benefits and minimize adverse effects.

Despite sharing a high degree of sequence identity and similar roles in immune cell recruitment, CXCR1 and CXCR2 exhibit distinct biological functions [135,162]. CXCR1 is predominantly associated with neutrophil activation and may influence specific pro-tumourigenic processes [163], whereas CXCR2 has a broader expression profile across different types of immune cells, including neutrophils, monocytes, and T-cells, and has been implicated in a wider range of tumourigenic processes, such as angiogenesis and metastasis. Thus, it is pivotal to identify isoform-specific inhibitors.

The choice between targeting either or both CXCR1/2 receptors depend on the specific tumourigenic pathways active in a given patient’s PCa. Inhibitors with a high selectivity for CXCR1 might provide benefits in scenarios where neutrophil-driven processes are predominant [163], whereas CXCR2 inhibitors could be more effective in a broader immune-modulatory context [63]. The lack of absolute specificity can be partially mitigated by using inhibitors in combination with ADT or ASIs, which may yield a synergistic therapeutic effect by attenuating the compensatory pathways that contribute to treatment resistance [139]. Ultimately, the decision on which receptors to target must be informed by a thorough understanding of tumour biology and of the molecular drivers of the disease in each individual patient. This precision medicine approach requires robust biomarkers to guide the selection of CXCR1/2 inhibitors, ensuring that the right drug is matched to the right patient, increasing the chances of enhancing both the specificity and efficacy of treatment.

The ongoing development of more selective CXCR1 and CXCR2 inhibitors, coupled with a deepening understanding of their roles in PCa pathophysiology, promises to refine this therapeutic strategy. However, further clinical studies are needed to elucidate the optimal use of these inhibitors in personalized treatment regimens. One of the major challenges with existing inhibitors remains the pharmacokinetics and dosing, as the inhibitors often exhibit high protein binding, complicating their exposure in patients. Additionally, there are concerns regarding neutrophil suppression in healthy patients, which has been observed in clinical trials with CXCR2 inhibitor AZD5069 [59]. Although this neutropenia did not result in febrile neutropenia, cautious dosing may have limited the optimal modulation of the signalling axis and limited efficacy. Addressing these pharmacokinetic issues and optimizing dosing strategies will be critical for the effective use of CXCR1/2 inhibitors in PCa treatment moving forward.

### 6.3. Side Effects

The potential for side effects, such as neutropenia [59], gastrointestinal disturbances, dermatologic effects (skin rashes), and other general effects (fatigue and malaise), remains a concern with CXCR1/2 inhibitors [164,165,166]. However, these are not the only adverse effects associated with CXCR1/2 inhibitors. While every treatment carries risks of adverse reactions, these must be weighed against the therapeutic gains, emphasizing the importance of patient-specific considerations and vigilant monitoring during and after treatment.

## 7. Discussion and Conclusions

This review critically assesses the role of IL-8 and its receptors, CXCR1 and CXCR2, in the progression of PCa, particularly in CRPC and mCRPC. A significant focus of this review is the central role of the TME and IME in PCa progression, where IL-8-driven inflammatory pathways are key contributors to the disease’s aggressiveness and resistance to conventional therapies. The complex interaction between cancer cells and the IME and TME underlie the intricate network that drives tumourigenesis and metastasis.

Current therapeutic approaches, such as ADT, ASIs, and chemotherapeutics, are limited by their eventual development of resistance. This review brings to light the necessity for novel therapeutic strategies that can circumvent or delay resistance mechanisms by targeting both the tumour cells and the TME and IME. The efficacy of targeting IL-8 signalling pathways, as evidenced by clinical trials like the ACE trial, points towards a promising improvement in PCa treatment, especially when combined with existing therapies such as ASI enzalutamide.

This review elucidates the multifaceted role of IL-8 and its receptors in PCa, emphasizing their involvement in the TME and IME with their contribution to disease progression and treatment resistance. Modulating the IL-8 signalling pathways holds significant therapeutic potential. The combination of CXCR1 and CXCR2 inhibition with standard treatments has shown promise in enhancing treatment efficacy and managing drug resistance. However, challenges such as specificity, pharmacokinetics, and potential side effects warrant careful consideration in clinical applications. Pharmacokinetic challenges, including high protein binding and the resultant difficulty in achieving optimal drug exposure, need to be addressed to improve the effectiveness of these inhibitors. Additionally, the potential for neutrophil suppression, while not causing febrile neutropenia, still requires attention to avoid undermining the therapeutic benefits.

## 8. Future Directions

As we look to the future, several key areas of research and clinical application emerge from our understanding of the role of IL-8 and its receptors in PCa. A primary focus remains on the refinement of therapeutic strategies to improve patient outcomes. This entails developing novel inhibitors or combinational therapies that can more effectively disrupt the IL-8 signalling pathways, thereby improving specificity, and reducing potential side effects. Moving towards a greater inhibition of the CXCR2 signalling axis, particularly in combination with ADT or ASIs, holds significant promise.

A deeper understanding of the molecular mechanisms underlying PCa progression, particularly the roles of IL-8 and its receptors in different stages of the disease, is crucial. Such knowledge can inform the development of targeted therapies and predictive biomarkers for treatment response. Access to tissues following treatment at various doses and combinations will ensure therapeutic interventions are appropriate tailored for maximum efficacy.

Further, the exploration of TME and IME dynamics, including the interaction of cancer cells with immune cells and other microenvironmental factors, remains essential. These research avenues will uncover new targets and lead to the development of more comprehensive treatment strategies. Addressing peripheral neutrophil challenges is particularly important, as a high polymorphonuclear (PMN)-to-T-cell ratio is associated with poor PCa prognosis. This suggests that PCa tumours may be cross-talking with the bone marrow, exerting a broader influence on the myeloid network.

And finally, given the heterogeneity of PCa, more personalized approaches are needed. Tailored therapies based on individual patient profiles, genetic makeup, and disease characteristics offer the most promise for future treatments. Future research should focus on refining these therapeutic strategies through a better understanding of the molecular mechanisms underlying IL-8 signalling in PCa, optimizing dosing regimens, and developing robust biomarkers to guide treatment. By integrating these strategies, the modulation of IL-8 signalling pathways can potentially transform the therapeutic landscape of PCa, offering improved outcomes and prolonged survival for patients.

## Figures and Tables

**Table 1:** Comparison of IL-8 ligands and CXCR1/CXCR2 expression in humans and mice.

Species	IL-8 Ligands	CXCR1/CXCR2 Ligands	CXCR1 Expression	CXCR2 Expression
**Human**	IL-8 (CXCL8)	CXCR1: CXCL6, CXCL8 CXCR2: CXCL1, CXCL2, CXCL3, CXCL5, CXCL6, CXCL7, CXCL8	Yes	Yes
**Mouse**	KC (CXCL1), MIP-2 (CXCL2), Gro-alpha (CXCL1)	CXCR1: CXCL6 CXCR2: CXCL1, CXCL2, CXCL3, CXCL5, CXCL6	No	Yes

Abbreviations: CXCL1: C-X-C chemokine ligand 1; CXCL2: C-X-C chemokine ligand 2; CXCL3: C-X-C chemokine ligand 3; CXCL5: C-X-C chemokine ligand 5; CXCL6: C-X-C chemokine ligand 6; CXCL7: C-X-C chemokine ligand 7; CXCL8: C-X-C chemokine ligand 8; CXCR1: C-X-C chemokine receptor type 1; CXCR2: C-X-C chemokine receptor type 2; Gro-alpha: chemokine growth-regulated protein alpha; IL-8: interleukin-8; KC: mouse surrogate C-X-C chemokine ligand 8; MIP-2: macrophage inflammatory protein 2.

**Table 2:** Overview of current inhibitors that target IL-8 or its receptors CXCR1 and/or CXCR2 in pre-clinical and clinical development.

Inhibitor	Clinical Development		CXCR1	CXCR2	IL-8
Navarixin (SCH-527123)	Phase 2	COPD Asthma Psoriasis Solid tumours			
Reparixin	Phase 3 Pneumonia	Pneumonia Acute respiratory distress syndrome Diabetes mellitus Solid tumours			
SB225002					
SB265610					
SX-682	Phase 1 and 2	Myelodysplastic syndromes Solid tumours			
AZD5069	Phase I2 Asthma Phase 1 and 2 mCRPC	Asthma Brochiectasis Solid tumours Pancreatic cancer HCC HNSCC			
Danirixin (GSK1325756)	Phase 1/2	COPD Viral disease			
Benzoylaconitine					
Pectolinarin					
Auraotene					
CXCR2 antagonist 4					
DF2726A					
BMS-986253 (HuMax-IL8)	Phase 1/2	MDS Solid tumours			

Abbreviations: COPD: chronic obstructive pulmonary disease; CXCR1: C-X-C chemokine receptor type 1; CXCR2: C-X-C chemokine receptor type 2; HCC: hepatocellular carcinoma; HNSCC: head and neck squamous-cell carcinoma; IL-8: interleukin 8; mCRPC: metastatic castrate-resistant prostate cancer; MDS: myelodysplastic syndrome.

**Table 3:** Overview of current compounds inhibiting IL-8 and CXCR1/2 interactions in completed or ongoing clinical trials in prostate cancer.

Drug Name	Trial Name	Target	Type	Last Reported Status	Summary	NCT Number	Results
**AZD5069** **(Astrazeneca)**	ACE	CXCR2	Small-molecule inhibitor	Phase 1 and 2: completed	AZD5069 in combination with enzalutamide.	NCT03177187	Results described in Section 4.3
**Navarixin** **(Merck)**		CXCR1/2	Small-molecule inhibitor	Phase 1 and 2: completed	Navarixin (MK-7123) in combination with pembrolizumab (MK-3475) in adults with selected advanced/metastatic solid tumours.	NCT03473925	Results posted on clinical trials.gov
**BMS-986253 (Previously Humax IL-8)** **(Bristol-Myers Squibb)**	MAGIC-8	IL-8	mAb	Phase 1b/2: active, not recruiting	Nivolumab (anti-PD-1) or nivolumab plus BMS-986253 in combination with ADT using degarelix (LHRH antagonist) for men with hormone-sensitive prostate cancer and a rising prostate-specific antigen (PSA).	NCT03689699	No results posted
**Burixafor Hydrobromide**		CXCR4		Pilot study: completed	Investigate single-agent burixafor hydrobromide, docetaxel, and G-CSF. Burixafor hydrobromide, alone or in combination with G-CSF, is currently in Phase 2 testing for use as a hematopoietic stem cell (HSC) mobilization agent.	NCT02478125	No results posted

Abbreviations: ACE: Combination Study of AZD5069 and enzalutamide; ADT: androgen deprivation therapy; CXCR1: C-X-C chemokine receptor type 1; CXCR2: C-X-C chemokine receptor type 2; CXCR4: C-X-C chemokine receptor type 4; G-CSF: granulocyte colony stimulating factor; HSC: hematopoietic stem cell; IL-8: interleukin 8; LHRH: luteinising hormone-releasing hormone; mAb: monoclonal antibody; NCT; National Clinical Trial number; PD-1: programmed cell death protein 1; PSA: prostate-specific antigen.

## Data Availability

This review article synthesizes and analyzes information from previously published studies and does not present any new data. Consequently, there are no datasets generated or analyzed during the current study to be made available. All data discussed within this review can be accessed through the cited references.

## Abbreviations

ACE	Proof of Concept Phase I/II Trial of the CXCR2 Antagonist AZD5069, Administered in Combination with enzalutamide, in Patients with Metastatic Castration-Resistant Prostate Cancer
ADT	Androgen deprivation therapy
AKT	Protein kinase B
AR	Androgen receptor
ASIs	Androgen-signalling inhibitors
BSP	Bone sialoprotein
CAFs	Cancer-associated fibroblasts
CD3+ T-cells, CD20+ B-cells, NK cells	Immune cells including T-cells, B-cells, and natural killer cells
CRPC	Castration-resistant prostate cancer
CSCs	Cancer stem cells
CTLA-4	Cytotoxic T-Lymphocyte Antigen 4
CTLA-5	Cytotoxic T-Lymphocyte Antigen 5
CXCL8	Chemokine (C-X-C motif) Ligand 8 (IL-8)
CXCR1	C-X-C chemokine receptor type 1
CXCR2	C-X-C chemokine receptor type 2
ECM	Extracellular matrix
ECs	Endothelial cells
EGF	Epidermal growth factor
EMT	Epithelial–mesenchymal transition
ENA-78	Epithelial-derived neutrophil-activating peptide 78
FDA	U.S. Food and Drug Administration
FGF	Fibroblast growth factor
GEMMs	Genetically engineered mouse model
Gleason score	A grading system for the histological patterns of prostate cancer
GPCR	G protein-coupled receptor
HCC	Hepatocellular carcinoma
hCXCR1 K1	Human CXCR1 knock-in
IGF	Insulin-like growth factor
IL-6	Interleukin-6
IL-8	Interleukin-8
IR	Ionizing radiation
JAK2	Janus kinase
KDM5C	Lysine (K)-specific Demethylase 5C
MAPK	Mitogen-activated protein kinase
mCRPC	Metastatic castration-resistant prostate cancer
MDSCs	Myeloid-derived suppressor cells
MMPs	Matrix metalloproteinases
MSCs	Mesenchymal stem cells
mTOR	Mammalian Target of Rapamycin
NEPC	Neuroendocrine prostate cancer
NF-kB	Nuclear Factor Kappa B
NK Cells	Natural killer cells
NLR	Neutrophil-to-lymphocyte ratio
P53	Tumour protein p53
PARP	Polyadenosine-diphosphate-ribose polymerase
PCa	Prostate cancer
PD-1	Programmed cell death protein 1
PDGF	Platelet-derived growth factor
PD-L1	Programmed death-ligand 1
PDXs	Patient-derived xenografts
PI3K/AKT	Phosphoinositide 3-kinase/protein kinase B
PKC	Protein kinase C
PMN	Polymorphonuclear
PSA	Prostate-specific antigen
PSMA	Prostate-specific membrane antigen
PTEN	Phosphatase and Tensin Homolog
RNA	Ribonucleic acid
RNT	Radionuclide therapy
STAT3	Single Transducer and Activation of Transcription
TAMs	Tumour-associated macrophages
TGF-β	Transforming growth factor-beta
TILs	Tumour-infiltrating lymphocytes
TME	Tumour microenvironment
TMPRSS-ERG	Transmembrane Protease, Serine 2—Erythroblast Transformation-Specific (ETS)-Related Gene
TNF	Tumour necrosis factor
TNFRSF14	Tumour necrosis factor Receptor Superfamily Member 14
TNF-α	Tumour necrosis factor-alpha
UBE2R2-AS1	Ubiquitin-Conjugating Enzyme E2 R2 Antisense RNA 1
VEGF	Vascular Endothelial Growth Factor
VEGF-A	Vascular Endothelial Growth Factor A

## References

[1] American Cancer Society (2023). Key Statistics for Prostate Cancer. https://www.cancer.org/cancer/types/prostate-cancer/about/key-statistics.html#:~:text=stage%20prostate%20cancer.-,Risk%20of%20prostate%20cancer,rare%20in%20men%20under%2040.

[2] Culp M.B., Soerjomataram I., Efstathiou J.A., Bray F., Jemal A. (2020). Recent Global Patterns in Prostate Cancer Incidence and Mortality Rates. Eur. Urol..

[3] Rawla P. (2019). Epidemiology of Prostate Cancer. World J. Oncol..

[4] Dearnaley D., Syndikus I., Mossop H., Khoo V., Birtle A., Bloomfield D., Graham J., Kirkbride P., Logue J., Malik Z. (2016). Conventional versus hypofractionated high-dose intensity-modulated radiotherapy for prostate cancer: 5-year outcomes of the randomised, non-inferiority, phase 3 CHHiP trial. Lancet Oncol..

[5] Hoffman K.E., Voong K.R., Levy L.B., Allen P.K., Choi S., Schlembach P.J., Lee A.K., McGuire S.E., Nguyen Q., Pugh T.J. (2018). Randomized Trial of Hypofractionated, Dose-Escalated, Intensity-Modulated Radiation Therapy (IMRT) versus Conventionally Fractionated IMRT for Localized Prostate Cancer. J. Clin. Oncol..

[6] Lee W.R., Dignam J.J., Amin M.B., Bruner D.W., Low D., Swanson G.P., Shah A.B., D’Souza D.P., Michalski J.M., Dayes I.S. (2016). Randomized Phase III Noninferiority Study Comparing Two Radiotherapy Fractionation Schedules in Patients with Low-Risk Prostate Cancer. J. Clin. Oncol..

[7] Sandhu S., Guo C., Hofman M.S. (2021). Radionuclide Therapy in Prostate Cancer: From standalone to combination PSMA theranostics. J. Nucl. Med..

[8] Ryan C.J., Smith M.R., Fizazi K., Saad F., Mulders P.F., Sternberg C.N., Miller K., Logothetis C.J., Shore N.D., Small E.J. (2015). Abiraterone acetate plus prednisone versus placebo plus prednisone in chemotherapy-naive men with metastatic castration-resistant prostate cancer (COU-AA-302): Final overall survival analysis of a randomised, double-blind, placebo-controlled phase 3 study. Lancet Oncol..

[9] Beer T.M., Armstrong A.J., Rathkopf D.E., Loriot Y., Sternberg C.N., Higano C.S., Iversen P., Bhattacharya S., Carles J., Chowdhury S. (2014). Enzalutamide in metastatic prostate cancer before chemotherapy. N. Engl. J. Med..

[10] Fizazi K., Tran N., Fein L., Matsubara N., Rodriguez-Antolin A., Alekseev B.Y., Özgüroğlu M., Ye D., Feyerabend S., Protheroe A. (2017). Abiraterone plus Prednisone in Metastatic, Castration-Sensitive Prostate Cancer. N. Engl. J. Med..

[11] Dai C., Heemers H., Sharifi N. (2017). Androgen Signaling in Prostate Cancer. Cold Spring Harb. Perspect. Med..

[12] Pezaro C. (2020). PARP inhibitor combinations in prostate cancer. Ther. Adv. Med. Oncol..

[13] Sathianathen N.J., Philippou Y.A., Kuntz G.M., Konety B.R., Gupta S., Lamb A.D., Dahm P. (2019). Taxane-based chemohormonal therapy for metastatic hormone-sensitive prostate cancer: A Cochrane Review. BJU Int..

[14] Baghban R., Roshangar L., Jahanban-Esfahlan R., Seidi K., Ebrahimi-Kalan A., Jaymand M., Kolahian S., Javaheri T., Zare P. (2020). Tumor microenvironment complexity and therapeutic implications at a glance. Cell Commun. Signal..

[15] Amit M., Baruch E., Nagarajan P., Gleber-Netto F., Rao X., Xie T., Akhter S., Adewale A., Islam S., Mattson B. (2023). Inflammation induced by tumor-associated nerves promotes resistance to anti-PD-1 therapy in cancer patients and is targetable by IL-6 blockade. Res. Sq..

[16] Armstrong C.W.D., Coulter J.A., Ong C.W., Maxwell P.J., Walker S., Butterworth K.T., Lyubomska O., Berlingeri S., Gallagher R., O’Sullivan J.M. (2020). Clinical and functional characterization of CXCR1/CXCR2 biology in the relapse and radiotherapy resistance of primary PTEN-deficient prostate carcinoma. NAR Cancer.

[17] Korbecki J., Bosiacki M., Chlubek D., Baranowska-Bosiacka I. (2023). Bioinformatic Analysis of the CXCR2 Ligands in Cancer Processes. Int. J. Mol. Sci..

[18] Aalinkeel R., Nair M.P., Sufrin G., Mahajan S.D., Chadha K.C., Chawda R.P., Schwartz S.A. (2004). Gene expression of angiogenic factors correlates with metastatic potential of prostate cancer cells. Cancer Res..

[19] Wang X., Xu F., Kou H., Zheng Y., Yang J., Xu Z., Fang Y., Sun W., Zhu S., Jiang Q. (2023). Stromal cell-derived small extracellular vesicles enhance radioresistance of prostate cancer cells via interleukin-8-induced autophagy. J. Extracell. Vesicles.

[20] Perdomo H.A.G., Zapata-Copete J.A., Sanchez A. (2018). Molecular alterations associated with prostate cancer. Cent. Eur. J. Urol..

[21] Holmes W.E., Lee J., Kuang W.J., Rice G.C., Wood W.I. (1991). Structure and functional expression of a human interleukin-8 receptor. Science.

[22] Murphy P.M., Tiffany H.L. (1991). Cloning of complementary DNA encoding a functional human interleukin-8 receptor. Science.

[23] Chen L., Fan J., Chen H., Meng Z., Chen Z., Wang P., Liu L. (2014). The IL-8/CXCR1 axis is associated with cancer stem cell-like properties and correlates with clinical prognosis in human pancreatic cancer cases. Sci. Rep..

[24] Singh J.K., Simões B.M., Howell S.J., Farnie G., Clarke R.B. (2013). Recent advances reveal IL-8 signaling as a potential key to targeting breast cancer stem cells. Breast Cancer Res..

[25] Sunaga N., Kaira K., Tomizawa Y., Shimizu K., Imai H., Takahashi G., Kakegawa S., Ohtaki Y., Nagashima T., Kasahara N. (2014). Clinicopathological and prognostic significance of interleukin-8 expression and its relationship to KRAS mutation in lung adenocarcinoma. Br. J. Cancer.

[26] Dahal S., Chaudhary P., Jung Y.S., Kim J.A. (2023). Megakaryocyte-Derived IL-8 Acts as a Paracrine Factor for Prostate Cancer Aggressiveness through CXCR2 Activation and Antagonistic AR Downregulation. Biomol. Ther..

[27] Cheng Y., Ma X.L., Wei Y.Q., Wei X.W. (2019). Potential roles and targeted therapy of the CXCLs/CXCR2 axis in cancer and inflammatory diseases. Biochim. Biophys. Acta Rev. Cancer.

[28] Seaton A., Scullin P., Maxwell P.J., Wilson C., Pettigrew J., Gallagher R., O’Sullivan J.M., Johnston P.G., Waugh D.J. (2008). Interleukin-8 signaling promotes androgen-independent proliferation of prostate cancer cells via induction of androgen receptor expression and activation. Carcinogenesis.

[29] Aurilio G., Cimadamore A., Mazzucchelli R., Lopez-Beltran A., Verri E., Scarpelli M., Massari F., Cheng L., Santoni M., Montironi R. (2020). Androgen Receptor Signaling Pathway in Prostate Cancer: From Genetics to Clinical Applications. Cells.

[30] Wilson C., Maxwell P.J., Longley D.B., Wilson R.H., Johnston P.G., Waugh D.J. (2012). Constitutive and treatment-induced CXCL8-signalling selectively modulates the efficacy of anti-metabolite therapeutics in metastatic prostate cancer. PLoS ONE.

[31] Asokan S., Bandapalli O.R. (2021). CXCL8 Signaling in the Tumor Microenvironment. Adv. Exp. Med. Biol..

[32] Saxena S., Singh R.K. (2021). Chemokines orchestrate tumor cells and the microenvironment to achieve metastatic heterogeneity. Cancer Metastasis Rev..

[33] Singh A.J., Gray J.W. (2021). Chemokine signaling in cancer-stroma communications. J. Cell Commun. Signal..

[34] Lopez-Bujanda Z.A., Haffner M.C., Chaimowitz M.G., Chowdhury N., Venturini N.J., Patel R.A., Obradovic A., Hansen C.S., Jacków J., Maynard J.P. (2021). Castration-mediated IL-8 promotes myeloid infiltration and prostate cancer progression. Nat. Cancer.

[35] Armstrong C.W., Coulter J.A., Ong C.W., Maxwell P.J., Walker S., Butterworth K.T., Lyubomska O., Berlingeri S., Gallagher R., O’Sullivan J.M. (2020). Targeting CXCR1 and CXCR2 to overcome radiotherapy resistance in PTEN-deficient prostate carcinoma. bioRxiv.

[36] Madan R.A., Palena C. (2021). Behind the IL-8 ball in prostate cancer. Nat. Cancer.

[37] Fousek K., Horn L.A., Palena C. (2021). Interleukin-8: A chemokine at the intersection of cancer plasticity, angiogenesis, and immune suppression. Pharmacol. Ther..

[38] Göbel A., Dell’Endice S., Jaschke N., Pählig S., Shahid A., Hofbauer L.C., Rachner T.D. (2021). The Role of Inflammation in Breast and Prostate Cancer Metastasis to Bone. Int. J. Mol. Sci..

[39] Hanahan D., Weinberg R.A. (2000). The hallmarks of cancer. Cell.

[40] Hanahan D., Weinberg R.A. (2011). Hallmarks of cancer: The next generation. Cell.

[41] Zhang H., Ye Y.L., Li M.X., Ye S.B., Huang W.R., Cai T.T., He J., Peng J.Y., Duan T.H., Cui J. (2017). CXCL2/MIF-CXCR2 signaling promotes the recruitment of myeloid-derived suppressor cells and is correlated with prognosis in bladder cancer. Oncogene.

[42] Xia M., Hyman B.T. (2002). GROalpha/KC, a chemokine receptor CXCR2 ligand, can be a potent trigger for neuronal ERK1/2 and PI-3 kinase pathways and for tau hyperphosphorylation-a role in Alzheimer’s disease?. J. Neuroimmunol..

[43] Long X., Ye Y., Zhang L., Liu P., Yu W., Wei F., Ren X., Yu J. (2016). IL-8, a novel messenger to cross-link inflammation and tumor EMT via autocrine and paracrine pathways (Review). Int. J. Oncol..

[44] Burger M., Hartmann T., Burger J.A., Schraufstatter I. (2005). KSHV-GPCR and CXCR2 transforming capacity and angiogenic responses are mediated through a JAK2-STAT3-dependent pathway. Oncogene.

[45] Wise A., Gearing K., Rees S. (2002). Target validation of G-protein coupled receptors. Drug Discov. Today.

[46] Ahuja S.K., Murphy P.M. (1996). The CXC chemokines growth-regulated oncogene (GRO) alpha, GRObeta, GROgamma, neutrophil-activating peptide-2, and epithelial cell-derived neutrophil-activating peptide-78 are potent agonists for the type B, but not the type A, human interleukin-8 receptor. J. Biol. Chem..

[47] Bozic C.R., Gerard N.P., von Uexkull-Guldenband C., Kolakowski L.F., Conklyn M.J., Breslow R., Showell H.J., Gerard C. (1994). The murine interleukin 8 type B receptor homologue and its ligands. Expression and biological characterization. J. Biol. Chem..

[48] Rovai L.E., Herschman H.R., Smith J.B. (1998). The murine neutrophil-chemoattractant chemokines LIX, KC, and MIP-2 have distinct induction kinetics, tissue distributions, and tissue-specific sensitivities to glucocorticoid regulation in endotoxemia. J. Leukoc. Biol..

[49] Asfaha S., Dubeykovskiy A.N., Tomita H., Yang X., Stokes S., Shibata W., Friedman R.A., Ariyama H., Dubeykovskaya Z.A., Muthupalani S. (2013). Mice that express human interleukin-8 have increased mobilization of immature myeloid cells, which exacerbates inflammation and accelerates colon carcinogenesis. Gastroenterology.

[50] Bizzarri C., Beccari A.R., Bertini R., Cavicchia M.R., Giorgini S., Allegretti M. (2006). ELR+ CXC chemokines and their receptors (CXC chemokine receptor 1 and CXC chemokine receptor 2) as new therapeutic targets. Pharmacol. Ther..

[51] Fahimi F., Alam M.J., Ang C., Adhyatma G.P., Xie L., Mackay C.R., Robert R. (2023). Human CXCR1 knock-in mice infer functional expression of a murine ortholog. J. Leukoc. Biol..

[52] Centenera M.M., Vincent A.D., Moldovan M., Lin H.M., Lynn D.J., Horvath L.G., Butler L.M. (2022). Harnessing the Heterogeneity of Prostate Cancer for Target Discovery Using Patient-Derived Explants. Cancers.

[53] Karkampouna S., La Manna F., Benjak A., Kiener M., De Menna M., Zoni E., Grosjean J., Klima I., Garofoli A., Bolis M. (2021). Patient-derived xenografts and organoids model therapy response in prostate cancer. Nat. Commun..

[54] Palanisamy N., Yang J., Shepherd P.D.A., Li-Ning-Tapia E.M., Labanca E., Manyam G.C., Ravoori M.K., Kundra V., Araujo J.C., Efstathiou E. (2020). The MD Anderson Prostate Cancer Patient-derived Xenograft Series (MDA PCa PDX) Captures the Molecular Landscape of Prostate Cancer and Facilitates Marker-driven Therapy Development. Clin. Cancer Res..

[55] Waseem M., Wang B.D. (2024). Organoids: An Emerging Precision Medicine Model for Prostate Cancer Research. Int. J. Mol. Sci..

[56] Medina S., Brockman A.A., Cross C.E., Hayes M.J., Mobley B.C., Mistry A.M., Chotai S., Weaver K.D., Thompson R.C., Chambless L.B. (2024). IL-8 Instructs Macrophage Identity in Lateral Ventricle Contacting Glioblastoma. bioRxiv.

[57] Harshman L.C., Wang V.X., Hamid A.A., Santone G., Drake C.G., Carducci M.A., DiPaola R.S., Fichorova R.N., Sweeney C.J. (2020). Impact of baseline serum IL-8 on metastatic hormone-sensitive prostate cancer outcomes in the Phase 3 CHAARTED trial (E3805). Prostate.

[58] Shang F.M., Li J. (2019). A small-molecule antagonist of CXCR1 and CXCR2 inhibits cell proliferation, migration and invasion in melanoma via PI3K/AKT pathway. Med. Clin..

[59] Guo C., Sharp A., Gurel B., Crespo M., Figueiredo I., Jain S., Vogl U., Rekowski J., Rouhifard M., Gallagher L. (2023). Targeting myeloid chemotaxis to reverse prostate cancer therapy resistance. Nature.

[60] Zhao B., Diao J., Li L., Kondo H., Li L., Hirono I. (2021). Molecular characterization and expression analysis of Japanese flounder (*Paralichthys olivaceus*) chemokine receptor CXCR2 in comparison with CXCR1. Dev. Comp. Immunol..

[61] Rani A., Dasgupta P., Murphy J.J. (2019). Prostate Cancer: The Role of Inflammation and Chemokines. Am. J. Pathol..

[62] Wei F., Wang D., Wei J., Tang N., Tang L., Xiong F., Guo C., Zhou M., Li X., Li G. (2021). Metabolic crosstalk in the tumor microenvironment regulates antitumor immunosuppression and immunotherapy resisitance. Cell. Mol. Life Sci..

[63] Ha H., Debnath B., Neamati N. (2017). Role of the CXCL8-CXCR1/2 Axis in Cancer and Inflammatory Diseases. Theranostics.

[64] Waugh D.J., Wilson C. (2008). The interleukin-8 pathway in cancer. Clin. Cancer Res..

[65] Murphy C., McGurk M., Pettigrew J., Santinelli A., Mazzucchelli R., Johnston P.G., Montironi R., Waugh D.J. (2005). Nonapical and cytoplasmic expression of interleukin-8, CXCR1, and CXCR2 correlates with cell proliferation and microvessel density in prostate cancer. Clin. Cancer Res..

[66] Khalaf K., Hana D., Chou J.T., Singh C., Mackiewicz A., Kaczmarek M. (2021). Aspects of the Tumor Microenvironment Involved in Immune Resistance and Drug Resistance. Front. Immunol..

[67] Bergers G., Benjamin L.E. (2003). Tumorigenesis and the angiogenic switch. Nat. Rev. Cancer.

[68] Zhou Z., Flesken-Nikitin A., Corney D.C., Wang W., Goodrich D.W., Roy-Burman P., Nikitin A.Y. (2006). Synergy of p53 and Rb deficiency in a conditional mouse model for metastatic prostate cancer. Cancer Res..

[69] Torres-Estay V., Carreño D.V., Francisco I.F.S., Sotomayor P., Godoy A.S., Smith G.J. (2015). Androgen receptor in human endothelial cells. J. Endocrinol..

[70] Fang B., Lu Y., Li X., Wei Y., Ye D., Wei G., Zhu Y. (2024). Targeting the tumor microenvironment, a new therapeutic approach for prostate cancer. Prostate Cancer Prostatic Dis..

[71] Dai J., Lu Y., Roca H., Keller J.M., Zhang J., McCauley L.K., Keller E.T. (2017). Immune mediators in the tumor microenvironment of prostate cancer. Chin. J. Cancer.

[72] Mao X., Xu J., Wang W., Liang C., Hua J., Liu J., Zhang B., Meng Q., Yu X., Shi S. (2021). Crosstalk between cancer-associated fibroblasts and immune cells in the tumor microenvironment: New findings and future perspectives. Mol. Cancer.

[73] Archer M., Dogra N., Kyprianou N. (2020). Inflammation as a Driver of Prostate Cancer Metastasis and Therapeutic Resistance. Cancers.

[74] Infanger D.W., Cho Y., Lopez B.S., Mohanan S., Liu S.C., Gursel D., Boockvar J.A., Fischbach C. (2013). Glioblastoma stem cells are regulated by interleukin-8 signaling in a tumoral perivascular niche. Cancer Res..

[75] Liu Q., Guo Z., Li G., Zhang Y., Liu X., Li B., Wang J., Li X. (2023). Cancer stem cells and their niche in cancer progression and therapy. Cancer Cell Int..

[76] Karantanos T., Corn P.G., Thompson T.C. (2013). Prostate cancer progression after androgen deprivation therapy: Mechanisms of castrate resistance and novel therapeutic approaches. Oncogene.

[77] Germann M., Wetterwald A., Guzmán-Ramirez N., van der Pluijm G., Culig Z., Cecchini M.G., Williams E.D., Thalmann G.N. (2012). Stem-like cells with luminal progenitor phenotype survive castration in human prostate cancer. Stem Cells.

[78] Wang G., Wang Z., Sarkar F.H., Wei W. (2012). Targeting prostate cancer stem cells for cancer therapy. Discov. Med..

[79] Du J., He Y., Li P., Wu W., Chen Y., Ruan H. (2018). IL-8 regulates the doxorubicin resistance of colorectal cancer cells via modulation of multidrug resistance 1 (MDR1). Cancer Chemother. Pharmacol..

[80] Iwasawa T., Yamauchi S., Fukunaga T., Orita H., Kato K. (2023). Novel subset of granulocytic MDSCs as immunosuppressive regulators and therapeutic targets in gastric cancer. Cancer Res..

[81] Li K., Shi H., Zhang B., Ou X., Ma Q., Chen Y., Shu P., Li D., Wang Y. (2021). Myeloid-derived suppressor cells as immunosuppressive regulators and therapeutic targets in cancer. Signal Transduct. Target. Ther..

[82] Gabrilovich D.I., Nagaraj S. (2009). Myeloid-derived suppressor cells as regulators of the immune system. Nat. Rev. Immunol..

[83] Garcia A.J., Ruscetti M., Arenzana T.L., Tran L.M., Bianci-Frias D., Sybert E., Priceman S.J., Wu L., Nelson P.S., Smale S.T. (2014). Pten null prostate epithelium promotes localized myeloid-derived suppressor cell expansion and immune suppression during tumor initiation and progression. Mol. Cell. Biol..

[84] Hellsten R., Lilljebjörn L., Johansson M., Leandersson K., Bjartell A. (2019). The STAT3 inhibitor galiellalactone inhibits the generation of MDSC-like monocytes by prostate cancer cells and decreases immunosuppressive and tumorigenic factors. Prostate.

[85] Maxwell P.J., McKechnie M., Armstrong C.W., Manley J.M., Ong C.W., Worthington J., Mills I.G., Longley D.B., Quigley J.P., Zoubeidi A. (2022). Attenuating Adaptive VEGF-A and IL8 Signaling Restores Durable Tumor Control in AR Antagonist-Treated Prostate Cancers. Mol. Cancer Res..

[86] Papanikolaou S., Vourda A., Syggelos S., Gyftopoulos K. (2021). Cell Plasticity and Prostate Cancer: The Role of Epithelial-Mesenchymal Transition in Tumor Progression, Invasion, Metastasis and Cancer Therapy Resistance. Cancers.

[87] Valerie Odero-Marah O.H., Henderson V., Sweeney J. (2018). Epithelial-Mesenchymal Transition (EMT) and Prostate Cancer. Adv. Exp. Med. Biol..

[88] Cheaito K.A., Bahmad H.F., Hadadeh O., Saleh E., Dagher C., Hammoud M.S., Shahait M., Mrad Z.A., Nassif S., Tawil A. (2019). EMT Markers in Locally-Advanced Prostate Cancer: Predicting Recurrence?. Front. Oncol..

[89] Wang F., Zhao M., Jiang Y., Xia S., Sun D., Zhou D., Dong Z. (2023). LncRNA UBE2R2-AS1, as prognostic marker, promotes cell proliferation and EMT in prostate cancer. Histol. Histopathol..

[90] Lemster A.L., Sievers E., Pasternack H., Lazar-Karsten P., Klümper N., Sailer V., Offermann A., Brägelmann J., Perner S., Kirfel J. (2022). Histone Demethylase KDM5C Drives Prostate Cancer Progression by Promoting EMT. Cancers.

[91] David J.M., Dominguez C., Hamilton D.H., Palena C. (2016). The IL-8/IL-8R Axis: A Double Agent in Tumor Immune Resistance. Vaccines.

[92] Torrealba N., Rodríguez-Berriguete G., Fraile B., Olmedilla G., Martínez-Onsurbe P., Guil-Cid M., Paniagua R., Royuela M. (2017). Expression of several cytokines in prostate cancer: Correlation with clinical variables of patients. Relationship with biochemical progression of the malignance. Cytokine.

[93] McShane R., Arya S., Stewart A.J., Caie P.D., Bates M. (2021). Prognostic features of the tumour microenvironment in oesophageal adenocarcinoma. Biochim. Biophys. Acta Rev. Cancer.

[94] Seebacher N.A., Krchniakova M., Stacy A.E., Skoda J., Jansson P.J. (2021). Tumour Microenvironment Stress Promotes the Development of Drug Resistance. Antioxidants.

[95] Burgos-Panadero R., Lucantoni F., Gamero-Sandemetrio E., Cruz-Merino L., Álvaro T., Noguera R. (2019). The tumour microenvironment as an integrated framework to understand cancer biology. Cancer Lett..

[96] Kiefer J.A., Farach-Carson M.C. (2001). Type I collagen-mediated proliferation of PC3 prostate carcinoma cell line: Implications for enhanced growth in the bone microenvironment. Matrix Biol..

[97] Henke E., Nandigama R., Ergün S. (2019). Extracellular Matrix in the Tumor Microenvironment and Its Impact on Cancer Therapy. Front. Mol. Biosci..

[98] Jiang Y., Zhang H., Wang J., Liu Y., Luo T., Hua H. (2022). Targeting extracellular matrix stiffness and mechanotransducers to improve cancer therapy. J. Hematol. Oncol..

[99] Pillai S.R., Damaghi M., Marunaka Y., Spugnini E.P., Fais S., Gillies R.J. (2019). Causes, consequences, and therapy of tumors acidosis. Cancer Metastasis Rev..

[100] Winkler J., Abisoye-Ogunniyan A., Metcalf K.J., Werb Z. (2020). Concepts of extracellular matrix remodelling in tumour progression and metastasis. Nat. Commun..

[101] Hirz T., Mei S., Sarkar H., Kfoury Y., Wu S., Verhoeven B.M., Subtelny A.O., Zlatev D.V., Wszolek M.W., Salari K. (2023). Dissecting the immune suppressive human prostate tumor microenvironment via integrated single-cell and spatial transcriptomic analyses. Nat. Commun..

[102] Shackleton E.G., Ali H.Y., Khan M., Pockley G.A., McArdle S.E. (2021). Novel Combinatorial Approaches to Tackle the Immunosuppressive Microenvironment of Prostate Cancer. Cancers.

[103] Tonry C., Finn S., Armstrong J., Pennington S.R. (2020). Clinical proteomics for prostate cancer: Understanding prostate cancer pathology and protein biomarkers for improved disease management. Clin. Proteom..

[104] Rycaj K., Cho E.J., Liu X., Chao H.P., Liu B., Li Q., Devkota A.K., Zhang D., Chen X., Moore J. (2016). Longitudinal tracking of subpopulation dynamics and molecular changes during LNCaP cell castration and identification of inhibitors that could target the PSA-/lo castration-resistant cells. Oncotarget.

[105] Karavitakis M., Ahmed H.U., Abel P.D., Hazell S., Winkler M.H. (2011). Tumor focality in prostate cancer: Implications for focal therapy. Nat. Rev. Clin. Oncol..

[106] Baca S.C., Prandi D., Lawrence M.S., Mosquera J.M., Romanel A., Drier Y., Park K., Kitabayashi N., MacDonald T.Y., Ghandi M. (2013). Punctuated evolution of prostate cancer genomes. Cell.

[107] Cotter K., Rubin M.A. (2022). The evolving landscape of prostate cancer somatic mutations. Prostate.

[108] Yoosuf N., Navarro J.F., Salmén F., Ståhl P.L., Daub C.O. (2020). Identification and transfer of spatial transcriptomics signatures for cancer diagnosis. Breast Cancer Res..

[109] Wang Y., Ma S., Ruzzo W.L. (2020). Spatial modeling of prostate cancer metabolic gene expression reveals extensive heterogeneity and selective vulnerabilities. Sci. Rep..

[110] Siewe N., Friedman A. (2022). Combination therapy for mCRPC with immune checkpoint inhibitors, ADT and vaccine: A mathematical model. PLoS ONE.

[111] Al-Akhras A., Chehade C.H., Narang A., Swami U. (2024). PARP Inhibitors in Metastatic Castration-Resistant Prostate Cancer: Unraveling the Therapeutic Landscape. Life.

[112] Turnham D.J., Bullock N., Dass M.S., Staffurth J.N., Pearson H.B. (2020). The PTEN Conundrum: How to Target PTEN-Deficient Prostate Cancer. Cells.

[113] van Dessel L.F., van Riet J., Smits M., Zhu Y., Hamberg P., van der Heijden M.S., Bergman A.M., van Oort I.M., de Wit R., Voest E.E. (2019). The genomic landscape of metastatic castration-resistant prostate cancers reveals multiple distinct genotypes with potential clinical impact. Nat. Commun..

[114] Sfanos K.S., De Marzo A.M. (2012). Prostate cancer and inflammation: The evidence. Histopathology.

[115] Gonzalez H., Hagerling C., Werb Z. (2018). Roles of the immune system in cancer: From tumor initiation to metastatic progression. Genes Dev..

[116] Krueger T.E., Thorek D.L.J., Meeker A.K., Isaacs J.T., Brennen W.N. (2019). Tumor-infiltrating mesenchymal stem cells: Drivers of the immunosuppressive tumor microenvironment in prostate cancer?. Prostate.

[117] de Bono J.S., Guo C., Gurel B., De Marzo A.M., Sfanos K.S., Mani R.S., Gil J., Drake C.G., Alimonti A. (2020). Prostate carcinogenesis: Inflammatory storms. Nat. Rev. Cancer.

[118] Cha J.H., Chan L.C., Li C.W., Hsu J.L., Hung M.C. (2019). Mechanisms Controlling PD-L1 Expression in Cancer. Mol. Cell.

[119] Li D., Zhou X., Xu W., Chen Y., Mu C., Zhao X., Yang T., Wang G., Wei L., Ma B. (2023). Prostate cancer cells synergistically defend against CD8(+) T cells by secreting exosomal PD-L1. Cancer Med..

[120] Willsmore Z.N., Coumbe B.G.T., Crescioli S., Reci S., Gupta A., Harris R.J., Chenoweth A., Chauhan J., Bax H.J., McCraw A. (2021). Combined anti-PD-1 and anti-CTLA-4 checkpoint blockade: Treatment of melanoma and immune mechanisms of action. Eur. J. Immunol..

[121] Kim C.W., Chon H.J., Kim C. (2021). Combination Immunotherapies to Overcome Intrinsic Resistance to Checkpoint Blockade in Microsatellite Stable Colorectal Cancer. Cancers.

[122] Sena L.A., Denmeade S.R., Antonarakis E.S. (2021). Targeting the spectrum of immune checkpoints in prostate cancer. Expert. Rev. Clin. Pharmacol..

[123] Yu S., Xiong G., Zhao S., Tang Y., Tang H., Wang K., Liu H., Lan K., Bi X., Duan S. (2021). Nanobodies targeting immune checkpoint molecules for tumor immunotherapy and immunoimaging (Review). Int. J. Mol. Med..

[124] Fizazi K., Drake C.G., Beer T.M., Kwon E.D., Scher H.I., Gerritsen W.R., Bossi A., den Eertwegh A., Krainer M., Houede N. (2020). Final Analysis of the Ipilimumab Versus Placebo Following Radiotherapy Phase III Trial in Postdocetaxel Metastatic Castration-resistant Prostate Cancer Identifies an Excess of Long-term Survivors. Eur. Urol..

[125] Aubert N., Brunel S., Olive D., Marodon G. (2021). Blockade of HVEM for Prostate Cancer Immunotherapy in Humanized Mice. Cancers.

[126] Czernin J., Current K., Mona C.E., Nyiranshuti L., Hikmat F., Radu C.G., Lückerath K. (2021). Immune-Checkpoint Blockade Enhances (225)Ac-PSMA617 Efficacy in a Mouse Model of Prostate Cancer. J. Nucl. Med..

[127] Ruiz de Porras V., Pardo J.C., Notario L., Etxaniz O., Font A. (2021). Immune Checkpoint Inhibitors: A Promising Treatment Option for Metastatic Castration-Resistant Prostate Cancer?. Int. J. Mol. Sci..

[128] Yu X., Li C., Wang Z., Xu Y., Shao S., Shao F., Wang H., Liu J. (2024). Neutrophils in cancer: Dual roles through intercellular interactions. Oncogene.

[129] Jurcevic S., Humfrey C., Uddin M., Warrington S., Larsson B., Keen C. (2015). The effect of a selective CXCR2 antagonist (AZD5069) on human blood neutrophil count and innate immune functions. Br. J. Clin. Pharmacol..

[130] Kumar S., O’Malley J., Chaudhary A.K., Inigo J.R., Yadav N., Kumar R., Chandra D. (2019). Hsp60 and IL-8 axis promotes apoptosis resistance in cancer. Br. J. Cancer.

[131] Hu X., Zhang H. (2019). Doxorubicin-Induced Cancer Cell Senescence Shows a Time Delay Effect and Is Inhibited by Epithelial-Mesenchymal Transition (EMT). Med. Sci. Monit..

[132] Zhang H., Yu Q.L., Meng L., Huang H., Liu H., Zhang N., Liu N., Yang J., Zhang Y.Z., Huang Q. (2020). TAZ-regulated expression of IL-8 is involved in chemoresistance of hepatocellular carcinoma cells. Arch. Biochem. Biophys..

[133] Bilusic M., Heery C.R., Collins J.M., Donahue R.N., Palena C., Madan R.A., Karzai F., Marté J.L., Strauss J., Gatti-Mays M.E. (2019). Phase I trial of HuMax-IL8 (BMS-986253), an anti-IL-8 monoclonal antibody, in patients with metastatic or unresectable solid tumors. J. Immunother. Cancer.

[134] Dhayni K., Zibara K., Issa H., Kamel S., Bennis Y. (2022). Targeting CXCR1 and CXCR2 receptors in cardiovascular diseases. Pharmacol. Ther..

[135] Prajapati D.R., Molczyk C., Purohit A., Saxena S., Sturgeon R., Dave B.J., Kumar S., Batra S.K., Singh R.K. (2023). Small molecule antagonist of CXCR2 and CXCR1 inhibits tumor growth, angiogenesis, and metastasis in pancreatic cancer. Cancer Lett..

[136] Steele C.W., Karim S.A., Leach J.D.G., Bailey P., Upstill-Goddard R., Rishi L., Foth M., Bryson S., McDaid K., Wilson Z. (2016). CXCR2 Inhibition Profoundly Suppresses Metastases and Augments Immunotherapy in Pancreatic Ductal Adenocarcinoma. Cancer Cell.

[137] Jackstadt R., van Hooff S.R., Leach J.D., Cortes-Lavaud X., Lohuis J.O., Ridgway R.A., Wouters V.M., Roper J., Kendall T.J., Roxburgh C.S. (2019). Epithelial NOTCH Signaling Rewires the Tumor Microenvironment of Colorectal Cancer to Drive Poor-Prognosis Subtypes and Metastasis. Cancer Cell.

[138] Leslie J., Mackey J.B.G., Jamieson T., Ramon-Gil E., Drake T.M., Fercoq F., Clark W., Gilroy K., Hedley A., Nixon C. (2022). CXCR2 inhibition enables NASH-HCC immunotherapy. Gut.

[139] Adekoya T.O., Smith N., Kothari P., Richardson R.M. (2022). Abstract PO-134: Differential effects of CXCR1 and CXCR2 receptors on prostate tumorigenesis. Cancer Epidemiol. Biomark. Prev..

[140] Nanda J.S., Koganti P., Perri G., Ellis L. (2022). Phenotypic Plasticity—Alternate Transcriptional Programs Driving Treatment Resistant Prostate Cancer. Crit. Rev. Oncog..

[141] de Bono J.S., Oudard S., Ozguroglu M., Hansen S., Machiels J.P., Kocak I., Gravis G., Bodrogi I., Mackenzie M.J., Shen L. (2010). Prednisone plus cabazitaxel or mitoxantrone for metastatic castration-resistant prostate cancer progressing after docetaxel treatment: A randomised open-label trial. Lancet.

[142] Parker C., Nilsson S., Heinrich D., Helle S.I., O’Sullivan J.M., Fosså S.D., Chodacki A., Wiechno P., Logue J., Seke M. (2013). Alpha emitter radium-223 and survival in metastatic prostate cancer. N. Engl. J. Med..

[143] Caffo O., Wissing M., Bianchini D., Bergman A., Thomsen F.B., Schmid S., Yu E.Y., Bournakis E., Sella A., Zagonel V. (2020). Survival Outcomes From a Cumulative Analysis of Worldwide Observational Studies on Sequential Use of New Agents in Metastatic Castration-Resistant Prostate Cancer. Clin. Genitourin. Cancer.

[144] Lombard A.P., Liu L., Cucchiara V., Liu C., Armstrong C.M., Zhao R., Yang J.C., Lou W., Evans C.P., Gao A.C. (2018). Intra versus Inter Cross-resistance Determines Treatment Sequence between Taxane and AR-Targeting Therapies in Advanced Prostate Cancer. Mol. Cancer Ther..

[145] Beer T.M., Kwon E.D., Drake C.G., Fizazi K., Logothetis C., Gravis G., Ganju V., Polikoff J., Saad F., Humanski P. (2017). Randomized, Double-Blind, Phase III Trial of Ipilimumab Versus Placebo in Asymptomatic or Minimally Symptomatic Patients With Metastatic Chemotherapy-Naive Castration-Resistant Prostate Cancer. J. Clin. Oncol..

[146] Trump D. (2016). Ipilimumab versus placebo after radiotherapy in patients with metastatic castration-resistant prostate cancer that had progressed after docetaxel chemotherapy (CA184-043): A multicentre, randomised, double-blind, phase 3 trial. Urol. Oncol..

[147] Li Y., He Y., Butler W., Xu L., Chang Y., Lei K., Zhang H., Zhou Y., Gao A.C., Zhang Q. (2019). Targeting cellular heterogeneity with CXCR2 blockade for the treatment of therapy-resistant prostate cancer. Sci. Transl. Med..

[148] Culig Z., Puhr M. (2018). Interleukin-6 and prostate cancer: Current developments and unsolved questions. Mol. Cell. Endocrinol..

[149] Culig Z. (2021). Response to Androgens and Androgen Receptor Antagonists in the Presence of Cytokines in Prostate Cancer. Cancers.

[150] Calcinotto A., Spataro C., Zagato E., Di Mitri D., Gil V., Crespo M., De Bernardis G., Losa M., Mirenda M., Pasquini E. (2018). IL-23 secreted by myeloid cells drives castration-resistant prostate cancer. Nature.

[151] Krause W. (2023). Resistance to prostate cancer treatments. IUBMB Life.

[152] Debes J.D., Tindall D.J. (2004). Mechanisms of androgen-refractory prostate cancer. N. Engl. J. Med..

[153] Cai M., Song X.L., Li X.A., Chen M., Guo J., Yang D.H., Chen Z., Zhao S.C. (2023). Current therapy and drug resistance in metastatic castration-resistant prostate cancer. Drug Resist. Updat..

[154] Grossmann M.E., Huang H., Tindall D.J. (2001). Androgen receptor signaling in androgen-refractory prostate cancer. J. Natl. Cancer Inst..

[155] Ge R., Wang Z., Montironi R., Jiang Z., Cheng M., Santoni M., Huang K., Massari F., Lu X., Cimadamore A. (2020). Epigenetic modulations and lineage plasticity in advanced prostate cancer. Ann. Oncol..

[156] Wen Y.C., Liu Y.N., Yeh H.L., Chen W.H., Jiang K.C., Lin S.R., Huang J., Hsiao M., Chen W.Y. (2021). TCF7L1 regulates cytokine response and neuroendocrine differentiation of prostate cancer. Oncogenesis.

[157] Meng Z.W., Zhang L., Cai X.R., Wang X., She F.F., Chen Y.L. (2023). Author Correction: IL-8 is a novel prometastatic chemokine in intrahepatic cholangiocarcinoma that induces CXCR2-PI3K/AKT signaling upon CD97 activation. Sci. Rep..

[158] Deng F., Weng Y., Li X., Wang T., Fan M., Shi Q. (2021). Overexpression of IL-8 promotes cell migration via PI3K-Akt signaling pathway and EMT in triple-negative breast cancer. Pathol. Res. Pract..

[159] Guo Y., Zang Y., Lv L., Cai F., Qian T., Zhang G., Feng Q. (2017). IL-8 promotes proliferation and inhibition of apoptosis via STAT3/AKT/NF-κB pathway in prostate cancer. Mol. Med. Rep..

[160] Wilson C., Purcell C., Seaton A., Oladipo O., Maxwell P.J., O’Sullivan J.M., Wilson R.H., Johnston P.G., Waugh D.J. (2008). Chemotherapy-induced CXC-chemokine/CXC-chemokine receptor signaling in metastatic prostate cancer cells confers resistance to oxaliplatin through potentiation of nuclear factor-kappaB transcription and evasion of apoptosis. J. Pharmacol. Exp. Ther..

[161] Park G.Y., Pathak H.B., Godwin A.K., Kwon Y. (2021). Epithelial-stromal communication via CXCL1-CXCR2 interaction stimulates growth of ovarian cancer cells through p38 activation. Cell. Oncol..

[162] Sitaru S., Budke A., Bertini R., Sperandio M. (2023). Therapeutic inhibition of CXCR1/2: Where do we stand?. Intern. Emerg. Med..

[163] Xiong S., Dong L., Cheng L. (2021). Neutrophils in cancer carcinogenesis and metastasis. J. Hematol. Oncol..

[164] Hu N., Westra J., Rutgers A., der Meer B.D.-V., Huitema M.G., Stegeman C.A., Abdulahad W.H., Satchell S.C., Mathieson P.W., Heeringa P. (2011). Decreased CXCR1 and CXCR2 expression on neutrophils in anti-neutrophil cytoplasmic autoantibody-associated vasculitides potentially increases neutrophil adhesion and impairs migration. Arthritis Res. Ther..

[165] Zhong J., Zong S., Wang J., Feng M., Wang J., Zhang H., Xiong L. (2023). Role of neutrophils on cancer cells and other immune cells in the tumor microenvironment. Biochim. Biophys. Acta Mol. Cell Res..

[166] Armstrong A.J., Geva R., Chung H.C., Lemech C., Miller W.H., Hansen A.R., Lee J.S., Tsai F., Solomon B.J., Kim T.M. (2024). CXCR2 antagonist navarixin in combination with pembrolizumab in select advanced solid tumors: A phase 2 randomized trial. Investig. New Drugs.

